# A Novel Framework to Aid the Development of Design Space across Multi-Unit Operation Pharmaceutical Processes—A Case Study of *Panax Notoginseng* Saponins Immediate Release Tablet

**DOI:** 10.3390/pharmaceutics11090474

**Published:** 2019-09-13

**Authors:** Fei Sun, Bing Xu, Shengyun Dai, Yi Zhang, Zhaozhou Lin, Yanjiang Qiao

**Affiliations:** 1 Department of Traditional Chinese Medicine Analysis, School of Chinese Materia Medica, Guangdong Pharmaceutical University, Guangzhou 510006, China; sunfei2017@gdpu.edu.cn; 2 Research Center of Traditional Chinese Medicine Information Engineering, Beijing University of Chinese Medicine, Beijing 100029, China; daishengyun1228@163.com (S.D.); zhangyi@njucm.edu.cn (Y.Z.); 3 Beijing Key Laboratory of Traditional Chinese Medicine Manufacturing Process Control and Quality Evaluation, Beijing 100029, China; 4 Division of Chinese Materia Medica, National Institutes for Food and Drug Control, Beijing 100050, China; 5 Department of Pilot Test Base, Beijing Institute of Clinical Pharmacy, Beijing 100035, China; linzhaozhou@gmail.com

**Keywords:** design space, multi-unit operation processes, multivariate statistical analysis, multi-block partial least squares path model, Quality by Design

## Abstract

The fundamental principle of Quality by Design (QbD) is that the product quality should be designed into the process through an upstream approach, rather than be tested in the downstream. The keystone of QbD is process modeling, and thus, to develop a process control strategy based on the development of design space. Multivariate statistical analysis is a very useful tool to support the implementation of QbD in pharmaceutical process development and manufacturing. Nowadays, pharmaceutical process modeling is mainly focused on one-unit operations and system modeling for the development of design space across multi-unit operations is still limited. In this study, a general procedure that gives a holistic view for understanding and controlling the process settings for the entire manufacturing process was investigated. The proposed framework was tested on the *Panax Notoginseng* Saponins immediate release tablet (PNS IRT) production process. The critical variables and the critical units acting on the process were identified according to the importance of explaining the variability in the multi-block partial least squares path model. This improved understanding of the process by illustrating how the properties of the raw materials, the process parameters in the wet granulation and the compaction and the intermediate properties affect the tablet properties. Furthermore, the design space was developed to compensate for the variability source from the upstream. The results demonstrated that the proposed framework was an important tool to gain understanding and control the multi-unit operation process.

## 1. Introduction

In recent decades, pharmaceutical industries have made great contributions to human health by discovering and developing new drugs. However, they are still far behind other industries, such as semiconductors, food or chemical industries with regard to manufacturing technologies [1,2]. Traditionally, pharmaceutical industries are mainly based on experience and fixed procedures for product and process development, and product manufacturing [3]. This is partly due to the strict regulatory framework under which pharmaceutical industries operate their business. This situation has long term effects and prevents companies from investing in improvements and innovations in the pharmaceutical process.

In order to encourage pharmaceutical industries to develop and implement innovations, the USA Food and Drug Administration (FDA) launched Quality by Design (QbD) initiatives [4,5], which aims to create an effective and flexible environment to consistently deliver quality products without oversight regulation. QbD is defined as “a systematic approach to development that begins with predefined objectives and emphasizes product and process understanding and process control based on sound science and quality risk management” [4]. According to QbD principles, the quality should be “built into” but not “tested into” the product [5] through a comprehensive understanding of the relationships between the critical material attributes (CMAs), the critical process parameters (CPPs) and the critical quality attributes (CQAs), and through effective controls on the variability. The ultimate objective of QbD is to promote a faster product and process development and to increase the manufacturing flexibility and process robustness to reduce production costs.

**Figure 1:**
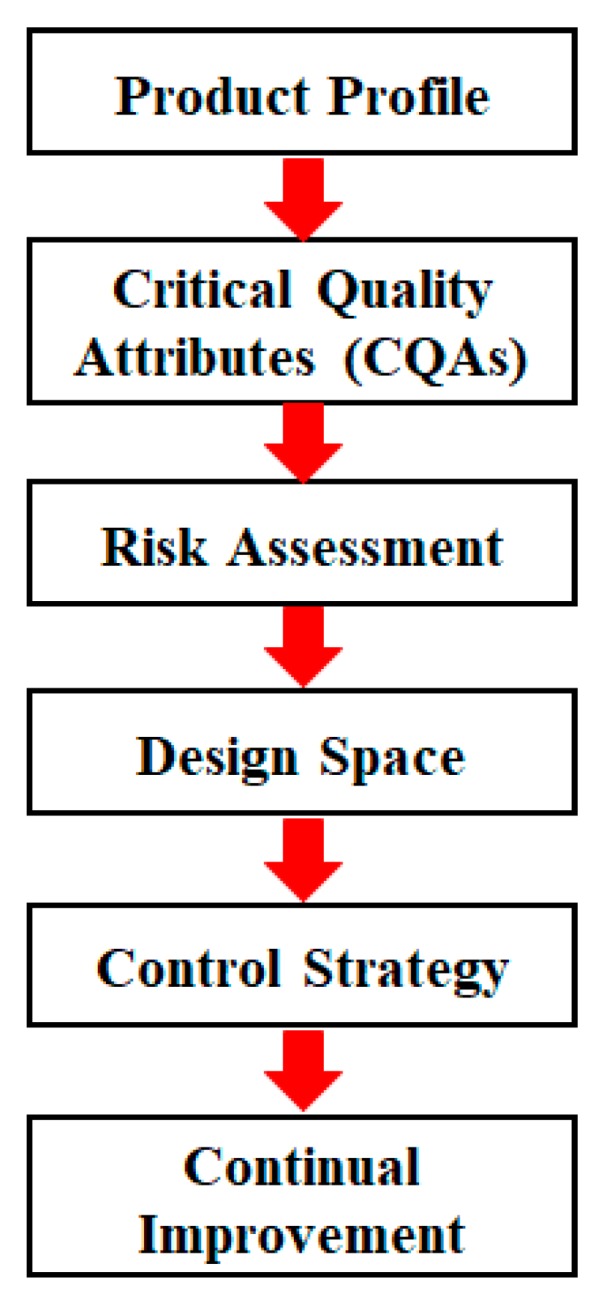
The steps in the product life cycle under the QbD framework.

QbD provides an enhanced approach to pharmaceutical development and manufacturing. In order to exploit the scientific and economic benefits of QbD, the practical implementation of QbD should go through several steps, as depicted in Figure 1. The design space is a keystone of QbD approaches and allows the development of a robust and stable manufacturing process with a science-based rationale [6]. The International Conference on Harmonistion of Technical Requirements for Registration of Pharmaceuticals for Human Use (ICH) Q8 guidance defines the design space as “the multidimensional combination and interaction of input variables and process parameters that have been demonstrated to provide assurance of quality” [4,7,8]. When the design space is established for a manufacturing process, working within the design space is not considered as a change. The concept of the design space is revolutionary for pharmaceutical development and improves the flexibility of pharmaceutical manufacturing. Although the design space is an optional feature of QbD approaches, its indisputable usefulness almost makes it a default feature of any QbD-based submissions [9,10,11,12,13]. To date, many studies have investigated the development of the design space. The design of experiment (DoE) is a basic tool to create design space as the DoE not only reduces the experimental work but also provides valuable information for quick decision making. Many published reports have highlighted the usefulness of experimental design to create the design space in small molecule pharmaceutical manufacturing such as extraction, crystallization, mixing and granulation [3,14,15,16,17]. Additionally, the formulation design space is essential in developing a robust product that delivers the desired quality over the product shelf life [18,19,20]. Induru et al. developed the formulation design space for a fast-dissolving tablet of diclofenac sodium with the full factorial design [21]. The need for further case studies on the application of design space to bioprocessing has also been emphasized. Jiang et al. described the development of a process design space for hydrophobic interaction chromatography purification of an Fc fusion protein [22]. Similar case studies on developing process design space for fermentation [23] and cell culture [24] have also been published. Recently, case studies on mapping design space for natural medicine have been published. Xu systematically discussed the framework to create a design space for Chinese herbal preparations [25]. Gong et al. proposed a novel method to develop a design space to improve the robustness of the ethanol precipitation process of the Danhong injection [26].

Today, new approaches for developing the design space for pharmaceutical processes are available including: (1) the dynamic design space [27,28], (2) the adaptive design space [29], and (3) the multi-unit design space. In this study, attention is focused on the development of the design space that spans multi-unit operation processes. In the abovementioned cases, the establishment of design space is mainly focused on single unit operations. However, the manufacturing of pharmaceutical products is usually based on several unit operations. Although advances in the development of the single unit design space has been significant, the interactions among the process parameters from different units and the influence of the upstream output on the downstream process have not been considered yet. So far, limited studies regarding integrating process system models into the implementation of QbD paradigms to establish the design space across multi units have been published.

The development of multi-unit design space requires building models of all critical units. The model used to describe the design space are based on first principle models, empirical models or hybrid models [30]. The first principle model, namely, the fundamental model, is derived when the underlying physical, chemical or biological phenomena are thoroughly understood and expressed as differential equations. Because first principle models provide clear representation of the relationships between the inputs and outputs of mechanisms, they are always desirable to assist in implementing QbD paradigms [31,32]. However, the main challenge of applying fundamental models is the high amount of effort and time required. Therefore, for junior scientists and engineers, the empirical model is a better choice as a considerable amount of process data can be collected from extensive experimental campaigns, on-going manufacturing processes and historical products that have already been developed. The empirical model is also called the data-driven model. Among the data-driven models, latent variable models (LVMs) [33] have been demonstrated to be useful in analyzing the experimental data and legacy [34,35,36,37,38]. The application of LVMs in product and process development, process understanding, and process monitoring and control are summarized by Tomba et al. [33]. Details of the theoretical background of LVMs and the relevant algorithms can be found elsewhere [39].

According to the summary reported by Troup et al. [40], pharmaceutical industries are showing an increasing interest in integrating mathematic modeling tools into manufacturing processes. The characteristics of LVMs could be useful to assist in modeling the pharmaceutical process system, as different forms of LVMs are suited to model different structures of manufacturing processes. For example, partial least squares (PLS) is always used to model the single unit operation, while multi-block partial least squares (MBPLS) is preferred to build a model of a processing system that covers a succession of units. Models of pharmaceutical process systems are useful for describing the relationships among the different process parameters in different units, so that a change in materials, or operation conditions at any point of the process can be related to intermediate and final product attributes. Modeling the process at the system level can be more feasible than modeling at the unit level, especially for the continuous manufacturing process, which is a system with multiple, highly integrated unit operations [41,42]. However, there are very few published studies on the design space of multi-unit operation processes with LVMs of the processing system.

Therefore, we proposed a general framework to develop the design space across multi-unit operation processes using LVMs in this study. The proposed approach was applied to a small scale experimental case study concerning the wet granulation and compaction process system for *Panax Notoginseng* Saponins immediate release tablet (PNS IRT). The aim of this study was to systematically use the existing methods to turn the process data into knowledge and to establish control strategies to deliver constant quality products.

## 2. Materials and Methods

### 2.1. Theory

#### 2.1.1. A Novel Framework to Develop the Design Space across Multi-Unit Operation Pharmaceutical Processes

**Figure 2:**
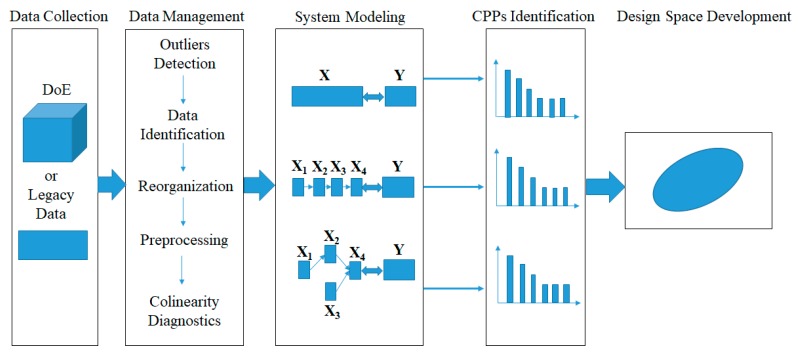
A novel framework to develop the design space across multi-unit operation pharmaceutical processes. DoE refers to design of experiment.

As displayed in Figure 2, a systematic procedure to establish a design space that spans multi-unit operation processes in a line includes the following activities: (1) data collection; (2) data preprocessing; (3) system modeling; (4) CPPs identification; and (5) design space development.

(1) Data Collection

The first step of the proposed framework is data collection. Generally, DoE is considered one of the most useful tools for the development of design space. Besides, a massive amount of data is generated and collected during the lifecycle of pharmaceutical products. Pharmaceutical companies can also benefit from better management of legacy data, from which useful information can be extracted for process understanding, process monitoring and process control.

(2) Data Management

Data management is crucial for the entire procedure despite its time-consuming nature. The objective of this step is to arrange the available data into different blocks that match the process flow-sheet as closely as possible. The main operations include outlier detection, identifying inputs and outputs of each unit operation, reorganizing the available data into different blocks, data preprocessing, and collinearity diagnostics.

Before analysis, outliers should be detected and eliminated from the data set as they may affect the performance of the process model in the subsequent analysis. Generally, the input variables of unit operation are material properties and manipulated process parameters whereas the output variables always represent the intermediate and final product properties and process measurements. After identification of the input and output variables, different data blocks are divided according to the unit operation or the variable types. Due to the dimensional differences in the collected variables and unhelpful information in the available data, it is essential to conduct data pretreatment before performing the subsequent analysis. Mean centering and unit variance are common preprocessing methods for the material and process data, while multiplicative scatter correction (MSC) and other smoothing methods [43] are usually used for spectral data. In addition, the collinearity among the variables should be evaluated to determine a suitable modeling algorithm to deal with this problem.

(3) System Modeling and CPPs Identification

The third step of the proposed framework is to model the pharmaceutical manufacturing process system to obtain a comprehensive understanding of the process. Under the QbD principle, the process is generally considered to be well understood when (1) all critical sources of variability are identified and explained; (2) variability is managed by the process; and (3) product quality attributes can be accurately and reliably predicted [44]. Therefore, the main aim of this step is to study how the variables in different units are related and interact, in order to illustrate how the downstream units or intermediate and final product properties are affected by the raw materials properties or process parameters in the upstream units. Hence, it is helpful to perform an exploratory analysis on each data block to understand the driving forces acting on each unit operation. Principal component analysis (PCA) is an effective tool to this end. Then the critical process units (CPUs) in the manufacturing line, as well as the CMAs or the CPPs in each unit can be determined through the process system model. The target can be realized through multi-block analysis such as multi-block principal component analysis (MBPCA) or MBPLS. Interpretation of the parameters in the system model can help to identify correlations among the variables of different blocks. Loadings of the model indicate which process parameters and attributes affect product quality and estimate their contribution to quality [45]. The variable importance in the projection (VIP) [46] helps identify the CPPs that contribute most to the product quality.

(4) Design Space Development

The final step of the framework is to develop a design space that spans the multi-unit operation process. An independent design space can be established for one or more-unit operations, and a single design space covering a series of successive unit operations is also acceptable. While a separate design space for each unit operation is easier to develop, an integrated design space that spans the entire process offers more operational flexibility because the type and location of the control action can be decided based on knowledge of the interactions of parameters between unit operations. Problems at the later stage can be anticipated and corrected at an early stage of the process.

#### 2.1.2. Partial Least Squares (PLS)

**Figure 3:**
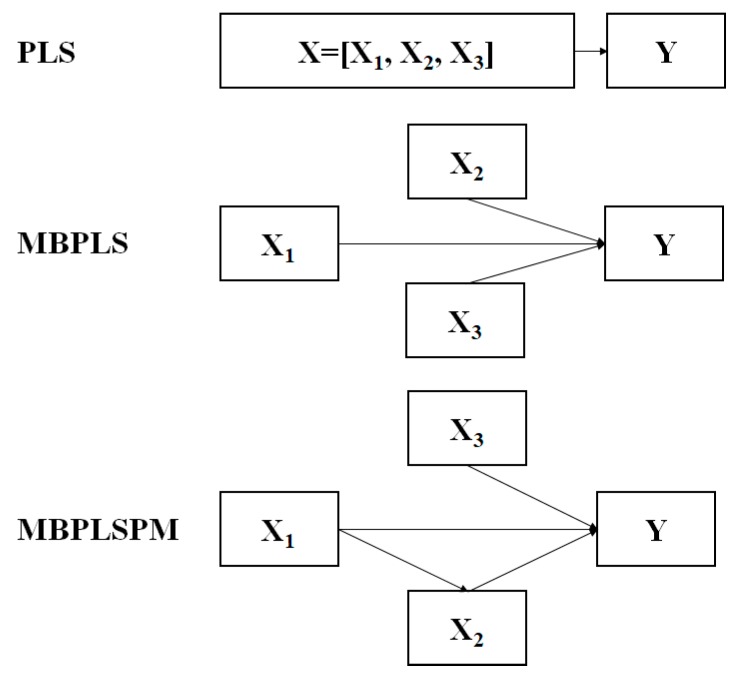
The schematic diagram of the partial least squares (PLS), multi-block partial least squares (MBPLS), and multi-block partial least squares path model (MBPLSPM) algorithm.

PLS is a LVM. The objective of PLS is to study the relationship between two matrixes, namely, the independent variables matrix (e.g., **X**) and response variables matrix (e.g., **Y**).The basic idea is that the PLS decomposes the two matrixes (e.g., **X** = [**X_1_**, **X_2_**, **X_3_**] and **Y** as seen in Figure 3) in a reduced latent variable space, in which the covariance between the projection of original samples is maximized.

**T** = **XW** (1)

**X** = **TP^T^** + **E** (2)

**Y** = **TQ^T^** + **F** (3)

In Equations (1)–(3), **W** is the weight of matrix **X**, **T** represents the scores of matrix **X**, **P** and **Q** stand for the loadings of matrix **X** and **Y**, respectively. **E** and **F** are the residuals of matrix **X** and **Y**, respectively.

#### 2.1.3. Multi-Block Partial Least Squares (MBPLS)

MBPLS is an extension of PLS to analyze the complex multivariate relationships among a set of data blocks [47]. Wold et al. originally developed and presented the main features of the MBPLS algorithm [48]. Later, two varieties of the MBPLS algorithm were reported. One used the block scores to calculate the loadings and residuals, while the other used the super scores. In this study, four data blocks (**X_1_**, **X_2_**, **X_3_**, and **Y**) were used to construct the MBPLS model (Figure 3) based on the later version. The MBPLS model is illustrated as follows:

**X_1_** = **T_s_P_1_^T^** + **E_1_** (4)

**X_2_** = **T_s_P_2_^T^** + **E_2_** (5)

**X_3_** = **T_s_P_3_^T^** + **E_3_** (6)

**Y** = **T_s_Q^T^** + **F** (7)

In Equations (4)–(7), **T_s_** refers to the super scores; **P_1_**, **P_2_**, and **P_3_** refer to the loadings of matrix **X_1_**, **X_2_**, and **X_3_**, respectively; **E_1_**, **E_2_**, and **E_3_** refer to the residuals of matrix **X_1_**, **X_2_**, and **X_3_**, respectively; **Q** refers to the loadings of matrix **Y**; **F** refers to the residuals of matrix **Y**.

#### 2.1.4. Multi-Block Partial Least Squares Path Model (MBPLSPM)

The MBPLSPM algorithm is a general form of the MBPLS algorithm. It was first proposed by Wangen and Kowalski [49]. This algorithm can be able to handle most types of relationships between different blocks and constitutes a significant advancement in the modeling of complex process systems [49]. The details of this algorithm were introduced in [49]. In this study, four data blocks (**X_1_**, **X_2_**, **X_3_**, and **Y**) were used to realize the MPLSPM. The pathway between different data blocks is shown in Figure 3. The MBPLSPM is assumed to be logically specified from left to right, where the left end blocks, namely, predictor blocks (e.g., **X_1_** and **X_3_**), only predict and the right end blocks (e.g., **Y**), namely, predictee blocks, are only predicted. The blocks in the middle, namely, interior blocks (e.g., **X_2_**), are both predictor blocks and predictee blocks.

The calculation procedure to construct the MBPLSPM is divided into a backward phase, where predictor vectors are calculated, and a forward phase for predictee vectors. The phase alternates until the predictee vector converges. The first step is to scale each block. Then, initialization of ***t*** and ***u*** vectors for **X_1_**, **X_2,_** and **X_3_** are selected.

In the backward phase, the scores of **X_1_**, **X_2_** and **X_3_** are calculated. Since **X_2_** and **X_3_** only predict **Y**, the ***t_X2_*** and ***t_X3_*** can be calculated as Equations (8)–(11):

**w_X2_** = **X_2′_u_Y_**, **w_X2_** = **w_X2_**/(**w_X2′_ w_X2_**)^1/2^ (8)

***t_X2_*** = **X_2_ w_X2_** (9)

**w_X3_** = **X_3′_u_Y_**, **w_X3_** = **w_X3_**/(**w_X3′_ w_X3_**)^1/2^ (10)

***t_X3_*** = **X_3_ w_X3_** (11)

where **w_X2_** and **w_X3_** are weights of **X_2_** and **X_3_**.

**X_1_** predicts both **X_3_** and **Y**. To calculate **t_X1_**, which predicts both blocks **X_3_** and **Y**, a superblock **U** that contains **u_X3_** and **u_Y_** is defined.

**U** = [**u_X3_**, **u_Y_**] (12)

***t_X1_*** is calculated as follows:

**c_U_** = **U’*t_X1_***, **c_U_** = **c_U_**/(**c_U_’ c_U_**)^1/2^ (13)

**u_U_** = **Uc_U_** (14)

**w_X1_** = **X_1′_u_U_**, **w_X1_** = **w_X1_**/(**w_X1′_ w_X1_**)^1/2^ (15)

***t_X1_*** = **X_1_w_X1_** (16)

In Equations (12)–(16), **c_U_** is the weight of **U**, **u_U_** is the score of **U**, **w_X1_** is the weight of **X_1_**.

In the forward phase, the scores of **X_3_** and **Y** are determined. **X_3_** is only predicted by **X_1_**, so **u_X3_** can be directly calculated as follows:

**c_X3_** = **X_3′_*t_X1_***, **c_X3_** = **c_X3_**/(**c_X3′_ c_X3_**)^1/2^ (17)

**u_X3_** = **X_3_ c_X3_** (18)

In Equations (17)–(18), **c_X3_** is the weights of **X_3_**.

**Y** is predicted by **X_1_**, **X_2_**, and **X_3_**. A superblock **T** is defined which consists of **t_X1_**, **t_X2_**, and **t_X3_**.

Assuming **w_T_** and **c_Y_** are the weights of **T** and **Y**, **u_Y_** can be calculated.

**w_T_** = **T’ u_Y_**, **w_T_** = **w_T_**/(**w_T_’ w_T_**)^1/2^ (19)

***t_T_*** = **T w_T_** (20)

**c_Y_** = **Y’ *t_T_***, **c_Y_** = **c_Y_**/(**c_Y_’ c_Y_**)^1/2^ (21)

**u_Y_** = **Y c_Y_** (22)

After completing one cycle of the backward and forward phase, **u_Y_** is tested for convergence within a desired precision (e.g., 10^−8^).

The loadings for predictor blocks (**p**) and predictee blocks (**q**) are calculated. As block scores ***t_X1_***, ***t_X2_*** and ***t_X3_*** are combined to calculate the super score **t_T_**, there are two methods, namely, the block score update method and the super score update method, to calculate the loadings. In this case, the former is used for calculating loadings of block **X_1_**, while the latter is used for block **X_2_** and **X_3_**.

**p_X1_** = **X_1′_*t_X1_***/(***t_X1′_ t_X1_***) (23)

**p_X2_** = **X_2′_*t_T_***/(***t_T_’ t_T_***) (24)

**p_X3_** = **X_3′_*t_T_***/(***t_T_’ t_T_***) (25)

**q_X3_** = **X_3′_ u_X3_**/(**u_X3′_ u_X3_**) (26)

**q_Y_** = **Y’ u_Y_**/(**u_Y_’ u_Y_**) (27)

The regression coefficients (**b**) are calculated for each block in the prediction.

**b_X1→U_** = **u_U_’ *t_X1_***/(***t_X1′_ t_X1_***) (28)

**b_T→Y_** = **u_Y_’ t_T_**/(**t_T_’ t_T_**) (29)

In Equations (28) and (29), **b_X1→U_** and **b_T→Y_** are used to predict **U** and **Y**, respectively.

For interior block **X_3_**, the regression coefficient used to determine the predictor and predictee part of **X_3_** is calculated in Equations (30) and (31).

**b_X1→X3_** = **c_U_(1) b_X1→U_**/(**c_U_’ c_U_**) (30)

**b_X3→Y_** = **w_T_(1) b_T→Y_**/(**w_T_’ w_T_**) (31)

Finally, assuming that **E_X1_**, **E_X2_**, **E_X3_**, and **E_Y_** are residuals of **X_1_**, **X_2_**, **X_3_**, and **Y**, respectively, the residuals are calculated for each block.

**E_X1_** = **X_1_** − ***t_X1_*p_X1′_** (32)

**E_X2_** = **X_2_** − ***t_X2_*p_X2′_** (33)

**E_Y_** = **Y** − **b_T→Y_*t_T_*c_Y_’** (34)

**E_X3_** = **X_3_** − (***s_X3_t_X3_*p_X3′_** + ***r_X3_*u_X3_c_X3′_**) (35)

In Equation (35), *r_X3_* = b_X1→X3_^2^/(b_X1→X3_^2^ + b_X3→Y_^2^), *s_X3_*^2^ = 1 − *r_X3_*^2^, **u_X3_** = b_X1→X3_ ***t_X1_***.

In the next cycle for calculating the following scores and loadings, **X_1_**, **X_2_**, **X_3_**, and **Y** are replaced by **E_X1_**, **E_X2_**, **E_X3_**, and **E_Y_**, respectively.

### 2.2. Materials

Eleven samples of PNS extracts were purchased from eight vendors including Ze Lang Pharmaceutical Co., Ltd. (Nanjing, China; lot No. ZL20141208, ZL20150120, ZL20150518, ZL20150524), San Sheng Pharmaceutical Co., Ltd. (Yunnan, China; lot No. 20140502 ), Yun Ke Pharmaceutical Co., Ltd. (Yunnan, China; lot No. 150906), Zhi Wu Pharmaceutical Co., Ltd. (Yunnan, China; lot No. HB20150308), Ben Cao Tian Gong Technology Co., Ltd. (Jiangxi, China; lot No. BCTG-0879), Yuan Cheng Gong Chuang Technology Co., Ltd. (Wuhan, China; lot No.98256), Ang Sheng Biological Technology Co., Ltd. (Shanxi, China; lot No. Usqzg151029) and Xi’an Hao Xuan Biotechnology Co., Ltd. (Shanxi, China; lot No. HXSQZZD150613). The different lots were defined as ZL1208, ZL0120, ZL0518, ZL0524, YNSS, YNYK, YNZW, BCTG, WHYC, SSAS and XAHX, respectively. The microcrystalline cellulose (MCC, Vivapur^®^ 101) was supplied by J. Rettenmaier & Söhne GmbH + CoKG (Rosenberg, Germany; lot No. 2610141813). The crospovidone (PVPP, XL-10) was purchased from ISP Chemicals, LLC (Calvert City, KY, USA; lot No. 0001873448). The magnesium stearate was purchased from Sinopharm Chemical Reagent Co., Ltd. (Shanghai, China; lot No. 20121010).

### 2.3. Design of Experiment

Several process parameters in the wet granulation and compaction process including pre-mixing time (A), impeller rate (B), binder amount (C), liquid additive rate (D), granulation time (E), lubrication time (F), and minimal punch tip separation distance (G) were chosen to perform the experimental design. The low- and high-level of each process parameter are shown in Table 1 and a D-optimal design was conducted with different lots of PNS extracts using Design Expert software (State-Ease Inc., Minneapolis, MN, USA). The details of the experiment are shown in Table 2.

### 2.4. PNS IRT Production Process

The PNS extracts (33.5%, *w*/*w*), MCC (60%, *w*/*w*) and PVPP (6%, *w*/*w*) were premixed in a high shear wet granulator with a volume of 2 L (SHK-4A, Xi’an Run Tian Pharmaceutical Machinery Co., Ltd., Xi’an, China). The mixed powders were granulated with 95% alcohol, which was added by a peristaltic pump. The wet granules were milled through a sieve manually and dried in a tray dryer (temperature at 80 °C). After that, the dried granules were lubricated with magnesium stearate (1%, *w*/*w*) in a 3-D blender with a 1 L volume (ZNW-10, Beijing Xing Shi Li He Technology Co., Ltd., Beijing, China). At last, the final blend was compressed into tablets using a rotary tablet press (ZP10, Shanghai Xin Yuan Pharmaceutical Machinery Co., Ltd., Shanghai, China).

### 2.5. Available Data

The available data were obtained from the designed experiments by processing different lots of PNS extracts under a set of different process operating conditions. The measurements were conducted on the input materials and the outputs of wet granulation and compaction. The measurement methods and results are shown in the Supplementary Materials. According to the flow sheet paradigm of the PNS IRT production process, the available data were arranged as seen in Table 3. Nine variables were measured to characterize the PNS extracts and the data were collected in Block **M** (11 × 9). The characteristics of PNS extracts included the bulk density (*D_bm_*), tapped density (*D_tm_*), particle size distribution (*D_10m_*, *D_50m_*, *D_90m_*, *Span_m_*), angle of repose (*AOR_m_*), Hausner ratio (*HR*_m_) and specific surface area (*SSA_m_*). During the granulation experiments, five variables were varied and arranged in Block **P_1_** (52 × 5). Granules from the wet granulation were dried and milled. All the experiments were conducted under the same drying and milling settings. The dried and milled granules were characterized by their bulk density (*D_b_*), tapped density (*D_t_*), particle size distribution (*D_10_*, *D_50_*, *D_90_*, *span*), moisture content (*MC*), angle of repose (*AOR*) and Hausner ratio (*HR*). The mean value of each variable across all samples was included corresponding to Block **X_2_** (52 × 9). The last step was compaction to get tablets. Before compaction, each run of granules was lubricated for a different time and then compressed into tablets by varying the minimal punch tip separation distance. The lubrication and compaction parameters were collected in Block **X_3_** (52 × 2). The tablets were measured by tensile strength (*TS*) and disintegration time (*DT*). These data were included in Block **Y** (52 × 2).

### 2.6. Multivariate Statistical Analysis

The MATLAB 7.8 software (Mathworks Inc., Natick, MA, USA) was used to perform PLS, MBPLS, and MBPLSPM. PLS Toolbox 2.1 (Eigenvector Research Inc., Manson, WA, USA) was used to perform the PLS regression. Other algorithms were realized using homemade programs.

## 3. Results and Discussion

### 3.1. Data Collection

All available data are described in detail in Section 2.5 of Materials and Methods.

### 3.2. Data Management

**Figure 4:**
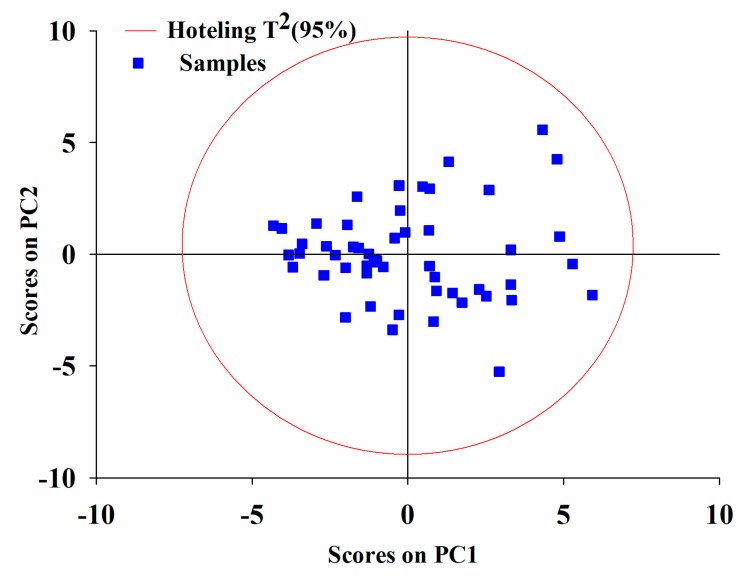
Score plot with the first two principal components (PCs) model for all available data.

All the available data were analyzed by PCA with 6 principal components (PCs) that accounted for 80% of the systematic variability. The score plot with the first two PCs was used to show the distribution of samples as displayed in Figure 4. The Hotelling T^2^ ellipse with 95% confidence [50] was calculated to identify potential outliers. As a result, none of the samples were beyond the threshold.

After outlier detection, the inputs and outputs for each unit operation were identified and all the available data were reorganized. Then all block data were preprocessed with the mean centering and unit variance. In order to support the model selection, the variance inflation factor (VIF) [51] was used to evaluate the collinearity of variables in each data block formed at different unit operations. The VIF was defined as Equation (36).

VIF*_i_* = 1/(1 − *r_i_*^2^) (36)

where *r_i_*^2^ refers to the coefficient of determination of multiple linear regression between *i*-th variable and other variables.

Table 4 reports the VIF value of each variable involved in each data block. Generally, the collinearity of variables is weak when the VIF value is less than 10. In this case, the variables that characterized the material and granule properties showed stronger collinearity, and the others were within the suggested limit. This indicated that multiple linear regression, such as polynomial regression was not recommended to estimate the process system to avoid strong collinearity.

### 3.3. Exploratory Analysis

The exploratory analysis was intended to identify the most important variables describing the variability in each data block, the correlations among them, and the distinctions between samples produced under different settings. In this case, PCA was used to analyze the data created in the previous step. It is known that scores and loadings are the main estimated parameters of the PCA model. The analysis of loadings and scores is crucial to make a practical application. Loadings are useful to understand the contribution of original variables to each PC and correlations among the variables. The variable with high loading absolute value has significant importance on the related PC. If the two variables have similar loadings on a PC, they are called to be correlated. If the loading absolute value is similar but the direction is opposite, they are called to be anti-correlated. Scores, namely PCs, are useful to visualize the samples in a low dimensional space as they explain most of the variability in the data. The distance between samples in the score plot reflects the similarity between samples in the original variable space. Two samples with similar scores are closely located in the score plot. By analyzing the PCA model parameters, an interpretation of the underlying phenomenon can be drawn.

#### 3.3.1. Material Properties (**M**)

A summary of the PCA model diagnostics including the eigenvalues, the explained variance per PC (*R*^2^) and cumulative explained variance per PC (*R*^2^*_cum_*) is shown in Table 5. Generally, the appropriate number of PCs should be considered to build the model. In the literature, several methods have been reported. In this case, the eigenvalue-greater-than-one rule was used to determine the number of PCs. Therefore, a PCA model was built by using the first two PCs, which explained 82.6% of the total variability in the input materials.

**Figure 5:**
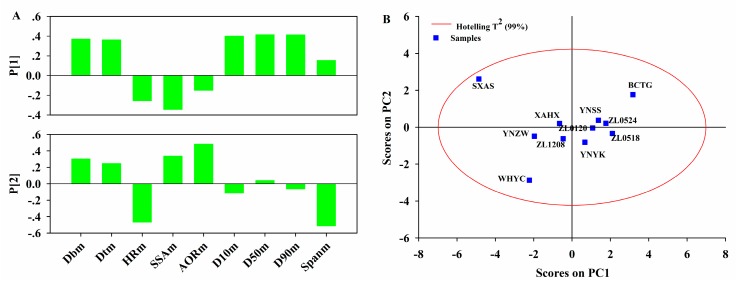
(**A**) Loading bar plots of PCA model on Block M; (**B**) Score plot of PCA model on Block **M**.

Figure 5A is the loading bar plot of the PCA model on Block **M**. It could be seen that *D_bm_*, *D_tm_*, *D_10m_*, *D_50m_ D_90m_*, and *SSA_m_* made great contributions to the first PC. *D_bm_*, *D_tm_*, *D_10m_*, *D_50m,_* and *D_90m_* had similar loading absolute values, so they were correlated. However, these variables were all inversely related to *SSA_m_*. This indicated that the PNS extracts with large particle size usually had high bulk and tapped density, and low specific surface area. Both *D_bm_* and *D_tm_* characterized the filling performance of the PNS extracts, and *D_10m_*, *D_50m_*, *D_90m_*, and *SSA*_m_ characterized the dimensions of the PNS extracts. Therefore, the physical meaning of the first PC can be summarized as the difference in filling performance and dimensions between different lots of PNS extracts. The variables that contributed most to the second PC were *AOR*_m_, *HR_m_*, and *Span*_m_. *AOR*_m_ and *HR_m_* were used to evaluate the powder flow-ability and *Span*_m_ was the homogeneity index. The second PC mainly described the difference in flow-ability and homogeneity between different lots of PNS extracts. The score plot of the PCA model on Block **M** is shown in Figure 5B. All lots of PNS extracts were within the Hotelling T^2^ ellipse with 99% confidence. YNZM, XAHX, Zl1208, ZL0120, ZL0518, ZL0524 and YNSS are projected close to each other in Figure 5B. This confirmed these lots had similar characteristics. The remaining lots, including SXAS, WHYC, and BCTG were located far from them. It was concluded that these three lots of PNS extract had differences in filling performance, dimensions, and flow-ability.

#### 3.3.2. Granulation Procedure ([**P_1_**, **X_2_**])

Granulation data including Block **P_1_** (wet granulation parameters) and **X_2_** (granule properties) were analyzed in order to understand how granulation parameters affected the granule properties. Therefore, a PCA model was built on a joint data block by concatenating the data block **P_1_** and **X_2_**. The diagnostics from the PCA model on the granulation data are listed in Table 6. It was observed that the first five PCs showed eigenvalues greater than 1 and thus the first five PCs, which accounted for 75.6% of total variance were used to build the model. The variability explained by the first two PCs was much higher than the others. The first two PCs captured a large fraction of the data variability. For this reason, the analysis was focused on the first two PCs.

**Figure 6:**
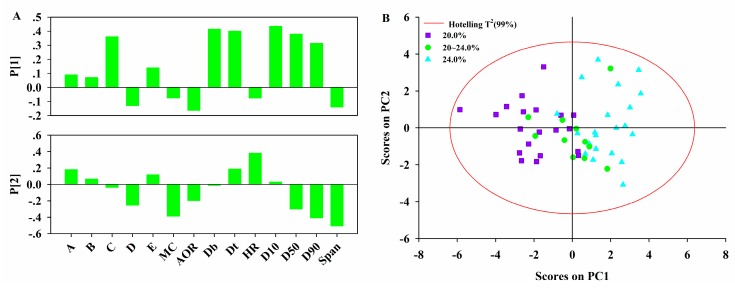
(**A**) Loading bar plots of PCA model on Block [**P_1_**, **X_2_**]; (**B**) Score plot of PCA model on Block [**P_1_**, **X_2_**]. A–E refer to the pre-mixing time, impeller rate, binder amount, liquid additive rate and granulation time, respectively.

Loading bar plots of the first two PCs are reported in Figure 6A. It was clearly seen that the first PC was driven by 6 variables of similar importance. *D_b_*, *D_t_*, *D_10_*, *D_50_*, *D_90_* and binder amount were correlated. This indicated that the first PC mainly represented the influence of binder amount on the granule particle size and density. Granules with large particle size and density should be obtained by wet granulation with more binder. The second PC mainly described *MC*, *HR,* and *Span*. *MC* usually affected the stability of the granules and controlling the *MC* of granules was critical to manufacturing desire quality tablets. *HR* and *span* were the indexes that characterize the granule flow-ability and homogeneity, respectively. A combined analysis of loading plots with the score plot in Figure 6B gave a deeper understanding of the results. Along the first PC direction, the granules produced with different binder amounts are marked with different symbols or colors. The lots processed with a low amount of binder are located in the region on the left of the score plot, while the lots processed with a high amount of binder fall mainly in the region on the right. The lots processed with a medium amount of binder are projected in the middle of score plot. This further confirmed that the binder amount used was positively correlated with the granule particle size and density.

#### 3.3.3. Compaction Procedure ([**X_3_**, **Y**])

The compaction data contained compaction parameters and tablet properties. Analysis of the compaction procedure was aimed at understanding how the compaction parameters affected the tablet properties. The data block X_3_ was combined with data block Y to form the joint data block [**X_3_**, **Y**]. The diagnostics of the PCA model on the joint data block are shown in Table 7. The first two PCs were enough to build the model as 78.6% of total variability was explained by two PCs.

**Figure 7:**
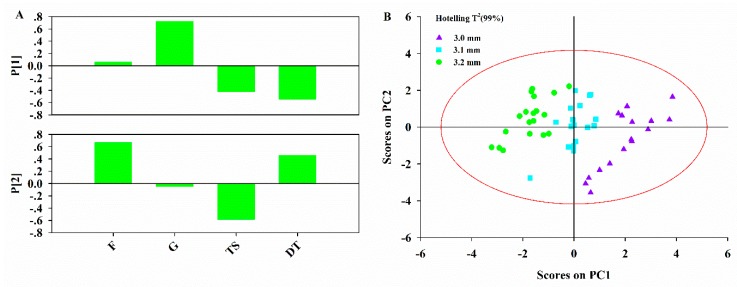
(**A**) Loading bar plots of PCA model on data block [**X_3_**, **Y**]; (**B**) Score plot of PCA model on data block [**X_3_**, **Y**]. F and G refer to lubrication time and minimal punch tip separation distance, respectively.

Analysis of loading plots (Figure 7A) indicated that the tablet properties were dominated by compaction parameters. The minimal punch tip separation distance was correlated with *TS* and *DT*. This meant that decreasing minimal punch tip separation distance resulted in tablets with higher *TS* and longer *DT*. The lubrication time was positively correlated with *DT* and negatively correlated with *TS*. From the viewpoint of the pharmaceutical engineer, the shorter lubrication time is expected to produce tablets with higher TS, but that disintegrate more quickly. The score plot (Figure 7B) shows that the first PC distinguished different lots produced under different compaction settings. The tablets produced under the higher minimal punch tip separation distance are located on the left of the score plot. Along the direction of the first PC, the *TS* of tablets increased as the minimal punch tip separation distance decreased.

### 3.4. System Modeling and CPPs Identification

The exploratory analysis aims to interpret the correlations among variables in a single data block at a unit operation scale. To achieve a working understanding that supports a sound design and efficient implementation, the manufacturing process should also be investigated at the system scale. The purpose of system modeling is to give a deeper understanding of the manufacturing process by studying the relationships between variables pertaining to different blocks and between blocks themselves. The advantage of LVMs is the fact that the model structure is transparent and able to explain the process in a straightforward way. LVMs such as PLS, MBPLS, and MBPLSPM are usually applied to model the pharmaceutical manufacturing process in a holistic way. In this case, the PLS, MBPLS, and MBPLSPM were applied to the PNS IRT production process and were compared to determine the optimal model. Then, the variables that have a significant influence on the final product quality can be identified based on the optimal model.

#### 3.4.1. Model Selection

For the PNS IRT production process, the raw material properties and wet granulation parameters have an effect on both the granule properties and tablet properties. So, these variables are organized in a single block (**X**_1_ = [**M**, **P_1_**]) to simplify the modeling procedure. In general, the prediction performance is primarily considered in model development. However, for the pharmaceutical process, the model interpretability is also of equal importance as the underlying information can be obtained to understand the mechanisms acting on the system. The amount of variability of original data explained by the model is usually used to represent the model interpretability, which is quantified by *R*^2^. The diagnostics of PLS, MBPLS, and MPLSPM model are shown in Table 8. The number of LVs for each model was selected by leave one out cross-validation. Three LVs were used to build the PLS, MBPLS and MBPLSPM models. In Table 8, *R*^2^_Xcum_ and *R*^2^_Ycum_ refer to the cumulative explained variance per LV for independent variables and response variables, respectively. *Q*^2^_Ycum_ refers to the cumulative explained variance per LV for modeling in the cross-validation. *Q*^2^ can be seen as a measure of the model predictive ability, and the prediction performance had no significant differences among different models, although the interpretability of the MBPLSPM was much higher than the others. The variation explained by the MBPLSPM for the independent variable matrix was 64.3%, and the model also captured a larger fraction of variability in the response variable matrix.

The pharmaceutical manufacturing process is a complex system. The aggregation and decomposition of this system across a hierarchy of appropriately chosen levels is the key to deal with the complexity. In this study, the data from different unit operations can be joined into a whole dataset and analyzed by the PLS model. Although PLS is a powerful modeling technique that relates one dataset to another, the relationship between different blocks cannot be represented. Hence, MBPLS and MBPLSPM are preferred to handle many types of pathway relationships between blocks in a complex system considering multiple data blocks in a single model. The interpretability of both MBPLS and MBPLSPM models is improved by keeping variables in separate blocks rather than a whole dataset. Compared with MBPLS, the MBPLSPM considers more complex pathway relationships between the blocks in the modeling procedure. Therefore, the MBPLSPM model explained the most variability in the original data.

#### 3.4.2. System Model

**Figure 8:**
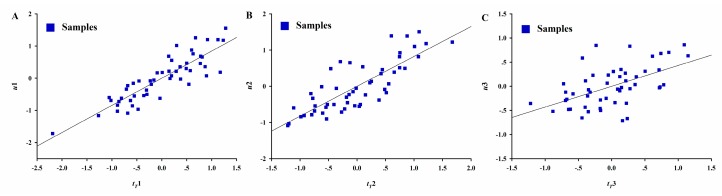
The relationships between the score of supermatrix **T** and that of matrix **Y** under the latent variables space of the MBPLSPM. (**A**) *t_T_1* vs. *u1*; (**B**) *t_T_2* vs. *u2*; (**C**) *t_T_3* vs. *u3*.

After the MBPLSPM was built on the PNS IRT process, the interpretation of model parameters in the original variable space or latent variable space was undertaken. The relationships between the scores of supermatrix T and that of tablet properties in the MBPLSPM are shown in Figure 8. It was observed that there was a linear relationship between the scores of the supermatrix and that of tablet properties under the first two LVs of the MBPLSPM. This indicated that the first two LVs described more variability than the third LV.

**Figure 9:**
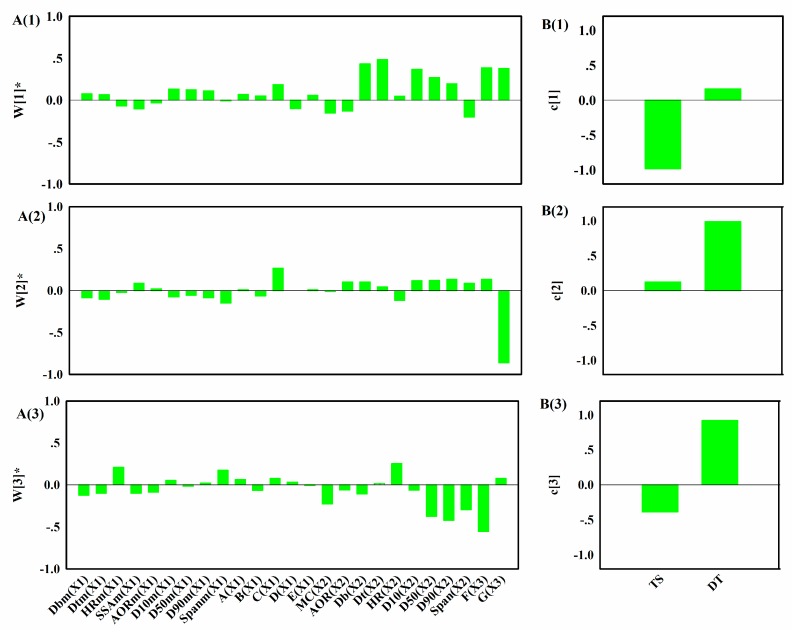
(**A**) Bar plots of the weights W* of [**X_1_**, **X_2_**, **X_3_**] in the MBPLSPM; (**B**) Bar plots of the loadings **Q** of **Y** in the MBPLSPM. A–F refers to pre-mixing time, impeller rate, binder amount, liquid additive rate, granulation time, lubrication time and minimal punch tip separation distance, respectively.

To understand how the variables in different blocks related and interacted, the loadings of the MBPLSPM model were analyzed. The bar plots of weights W* of matrix [**X_1_**, **X_2_**, **X_3_**], weighted on the variables are showed in Figure 9A. The bar plots of loadings **Q** of **Y**, which indicated the contribution of each variable in LVs are reported in Figure 9B.

The first LV mainly described the relationships between the *TS* and the variables in **X_2_** and **X_3_**, i.e., *D_b_*, *D_t_*, *D_10_*, *D_50_*, *D_90_* and compaction process parameters. There appeared to be positive correlations between the granule size and the granule density. As expected, large granules after compaction resulted in tablets with lower *TS*. Decreasing the lubrication time and minimal punch tip separation distance resulted in tablets with higher *TS*. The second LV showed that increasing the minimal punch tip separation distance led to tablets faster disintegration. The third LV showed that the granule size and lubrication time also contributed to the *DT*, which was an expected occurrence; tablets compressed with large granules and longer lubrication time may disintegrate faster.

#### 3.4.3. CPPs Identification

In order to manufacture the desired quality product, the critical process units (CPUs) and CPPs should be identified. In the PNS IRT production process the raw material properties, granulation process parameters, granule properties, and compaction process parameters have an impact on the tablet properties. It is necessary to rank the importance of different blocks to identify the CPUs. The importance of the *i*-th block in the projection can be evaluated by the block importance in the projection (BIP) index, which is defined as follows [52]:

(37) $$BIP_{i}=\sqrt{\frac{m\sum_{k=1}^{K}R_{Y,k}^{2}{\left(w_{{}_{i,k}}^{s}\right)}^{2}}{\sum_{k=1}^{K}R_{Y,k}^{2}}}$$

where *m* is the number of blocks considered, *R_Y,k_*^2^ is the variance of tablet properties explained by the *k*-th latent variable, and w*_i,k_*^s^ is the weight of *i*-th block on the *k*-th latent variable calculated from the MBPLSPM.

**Figure 10:**
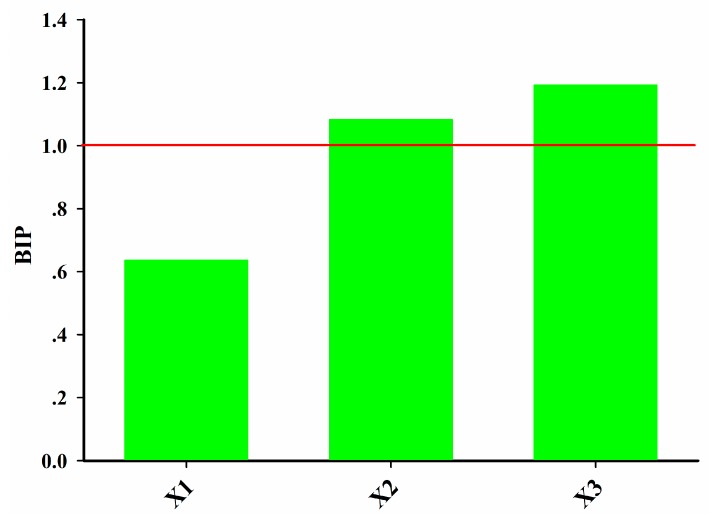
Block importance in the projection (BIP) index of each block in the MBPLSPM model.

The BIP index of each block is displayed in Figure 10. A threshold equal to 1 was used to assess whether a block was important or not in the prediction of tablet properties. In this study, the BIP values of two blocks, i.e., **X_2_** and **X_3_** were larger than 1. The granule properties and compaction process parameters contributed most to the tablet properties.

Once the CPUs are determined, the CMAs, the CQAs in the intermediate, and the CPPs are identified. The same type of index is calculated for the importance of each variable in the projection, namely, the VIP index, which is calculated as follows.

(38) $$VIP_{i}=\sqrt{\frac{n\sum_{k=1}^{K}R_{Y,k}^{2}{\left(w_{i,k}\right)}^{2}}{\sum_{k=1}^{K}R_{Y,k}^{2}}}$$

where *n* is the total number of variables considered, *R_Y,k_*^2^ is the variance of tablet properties explained by the *k*-th latent variable, while w_i,k_ is the weight of *i*-th variable on the *k*-th latent variable.

**Figure 11:**
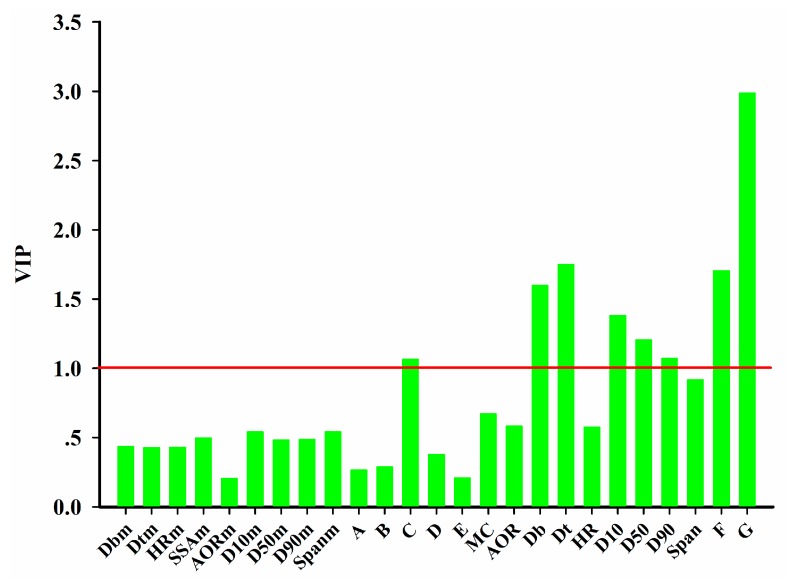
Variable importance in the projection (VIP) indexes of each variable in the MBPLSPM. A–F refers to pre-mixing time, impeller rate, binder amount, liquid additive rate, granulation time, lubrication time and minimal punch tip separation distance, respectively.

Figure 11 shows the VIP indexes of each variable in the MBPLSPM. The same threshold equal to 1 was used to identify the critical variables in the prediction of tablet properties. As seen in Figure 11, binder amount, *D_b_*, *D_t_*, *D_10_*, *D_50_*, *D_90_*, lubrication time and minimal punch tip separation distance were identified as the critical variables.

### 3.5. Design Space Development

The aim of the design space for the entire manufacturing process is to ensure that all CQAs of the tablets meet the quality targets when the process is run within the proposed design space. In this study, *TS* and *DT* were taken into consideration to develop the multi-unit process design space. The targets of each CQA were set up as: *TS* 3~5 MPa, *DT* < 5 min.

The CPPs that affected the CQAs included binder amount, *D_t_*, *D_b_*, *D_10_*, *D_50_*, *D_90_*, lubrication time and minimal punch tip separation distance. The CPPs covered the wet granulation and compaction process and an integrated design space can be developed to control the CQAs of tablets. As the granule density (*D_t_*, *D_b_*) and granule size (*D_10_*, *D_50_*, and *D_90_*) were correlated with binder amount, controlling the binder amount in the wet granulation resulted in desire granules. For this reason, binder amount, lubrication time and minimal punch tip separation distance were determined to be the final variables for developing the design space. The prediction formula of each CQA is shown below.

*TS* = 31.086 − 0.292 × Binder amount − 0.065 × Lubrication time − 6.333 × Minimal punch tip separation distance, *R*^2^ = 0.863, *R*^2^*_adj_* = 0.836.

*DT* = 584.813 + 0.885 × Binder amount + 0.540 × Lubrication time − 376.339 × Minimal punch tip separation distance − 0.023 × Binder amount × Lubrication time + 58.911 × Minimal punch tip separation distance × Minimal punch tip separation distance, *R*^2^ = 0.828, *R*^2^*_adj_* = 0.8096.

**Figure 12:**
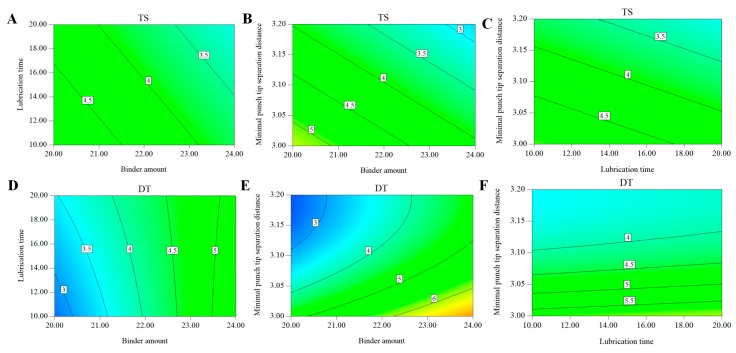
Contour plot showing the effects of the binder amount, lubrication time, and minimal punch tip separation distance on the tensile strength and disintegration time.

The coefficient of determination (*R*^2^) and the adjusted coefficient of determination (*R*^2^*_adj_*) for the prediction model of *TS* were 0.863, and 0.836, respectively, while the coefficient of determination and the adjusted coefficient of determination for the prediction model of DT were 0.828, and 0.8096, respectively. In addition, the analysis of variance was performed on the regression models. It was confirmed that the models were statistically significant with a *p* value <0.5. These results indicated that the established models were acceptable and suitable for building the design space. Based on the established regression models, the contour plots were constructed to visualize the effects of the binder amount, lubrication time and minimal punch tip separation distance on the DT and TS in Figure 12. It could be inferred from contour plots that increasing the binder amount, lubrication time, and the minimal punch tip separation distance would result in tablets with higher TS while decreasing the binder amount and lubrication time, and increasing the minimal punch tip separation distance would result in tablets with faster disintegration.

**Figure 13:**
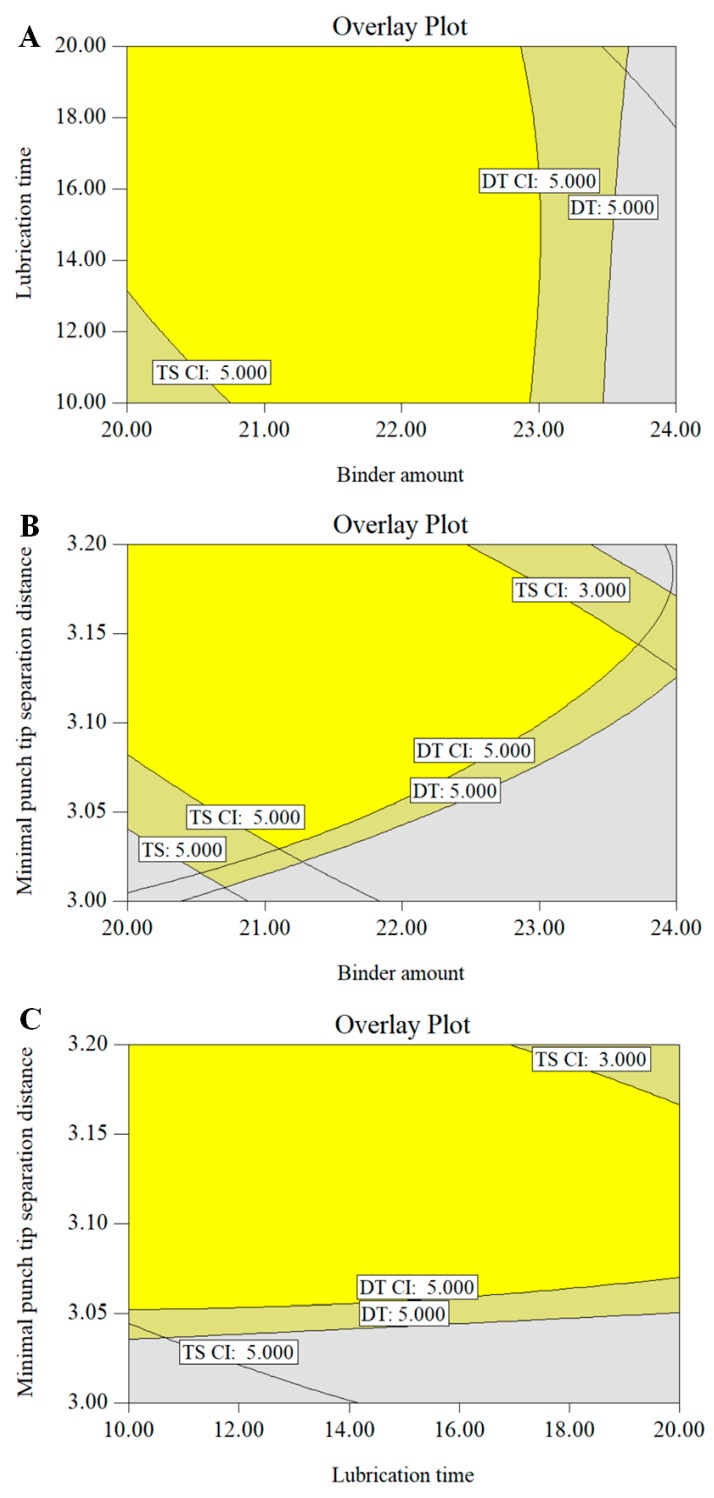
The design space for the PNS IRT production process; (**A**) binder amount vs. lubrication time; (**B**) binder amount vs. minimal punch tip separation distance; (**C**) lubrication vs. minimal punch tip separation distance.

A design space can be achieved for each CQA, and these separate design spaces are then overlapped. The integrated design space for the entire manufacturing process is proposed as seen in Figure 13. The yellow region in Figure 13A–C represented the proposed design space. The range of binder amount, lubrication time and minimal punch tip separation distance for the proposed design space were: a binder amount of 21–23%, lubrication time of 10–20 min, and a minimal punch tip separation distance of 3.1–3.2 mm. This indicated that the CPPs could be controlled in the acceptable range to produce the desired quality of tablets.

## 4. Conclusions

In this paper, a novel framework for the development of design space across multi-unit operation processes with multivariate statistical analysis is presented. The proposed strategy aims to formalize the application of LVMs to the experiment or legacy data to gain a systematic understanding and define a control strategy based on the design space. The procedure involves four main steps. The first step is data collection and data from designed experiments or legacy can be used. The next step is data management in which the data are reorganized in matrices corresponding to the process units. The third step is system modeling and CPPs identification. In this step, a systematic modeling method should be performed on the available data in order to define the relationships between the variables in different blocks and determine the critical variables or units affecting the quality attributes. In the last step, the design space is developed to control the product quality by compensating the variability from the upstream.

The proposed framework was successfully applied to an experimental case study concerning the PNS IRT production process. The MBPLSPM was built to show which units were most influential to the product quality and to show which variables were most correlated to the quality attributes. Binder amount, lubrication time and minimal punch tip separation distance, which covered the wet granulation and compaction processes were combined to develop the design space in order to meet the quality targets of tablets. The results confirmed that this approach was helpful to improve understanding of the process and offer considerable promise for setting up the process system.

## Figures and Tables

**Table 1:** Factors and levels of D-optimal design.

Factors	Levels	
	Low-Level	High-Level
A (min)	5	15
B (rpm)	400	600
C (%)	20	24
D (mL/min)	10	20
E (min)	3	5
F (min)	10	20
G (mm)	3.0	3.2

**Table 2:** D-optimal design for the *Panax Notoginseng* Saponins immediate release tablet (PNS) IRT production process with different lots of PNS extracts.

Run	Lot No.	Granulation					Compaction	
		A (min)	B (rpm)	C (%)	D (mL/min)	E (min)	F (min)	G (mm)
1	ZL0518	15	400	20	19.9	5	10	3.2
2	SXAS	5	410	22.8	18.9	4.3	20	3.0
3	XAHX	5	400	20	20	3	10	3.0
4	YNZW	8.8	500	24	10	5	10	3.1
5	ZL0524	15	400	24	20	5	20	3.0
6	WHYC	5	400	22.2	20	5	10	3.1
7	YNYK	8.1	500	20	17.5	3	10	3.1
8	BCTG	15	455	22	14.5	4.1	13.4	3.1
9	ZL0518	15	500	24	10	3	20	3.1
10	BCTG	13.4	500	24	10	5	20	3.0
11	YNZZ	5	400	24	20	3	15.9	3.2
12	ZL0518	5	400	22	10	5	10	3.2
13	ZL1208	5	400	20	10	5	14.8	3.1
14	ZL0524	5	500	24	20	3.9	20	3.1
15	YNZW	5	500	20	20	3	20	3.0
16	SXAS	15	500	22.1	20	3	20	3.2
17	SXAS	5	400	24	10	5	20	3.0
18	WHYC	15	400	20	20	3	20	3.1
19	ZL1208	15	400	24	10	4.2	20	3.2
20	BCTG	15	433	24	15.2	3	20	3.0
21	ZL0120	15	500	22.1	20	3	20	3.2
22	BCTG	10.75	400	24	14.3	5	20	3.1
23	ZL1208	5	434	21.6	15.1	3.6	14	3.1
24	YNSS	15	400	20	10	5	20	3.0
25	SXAS	5	400	20	12.9	3	20	3.2
26	BCTG	5	486	24	10	3	20	3.2
27	ZL0518	5	400	24	10	3	10	3.1
28	YNSS	5.7	441	20	20	5	20	3.2
29	YNSS	15	500	20	20	5	10	3.0
30	ZL0120	5	500	21.4	10	5	20	3.1
31	ZL0518	15	400	24	10	4.2	20	3.2
32	YNSS	15	400	24	20	3	10	3.1
33	YNYK	15	500	20	20	5	20	3.1
34	WHYC	15	464	24	20	5	10	3.2
35	ZL0120	7.05	500	21.2	19.7	5	14.6	3.1
36	ZL0518	8.5	500	21.6	10.3	3.8	13.7	3.2
37	YNSS	5	500	24	20	3	10	3.0
38	YNYK	15	400	20	10	3	10	3.1
39	XAHX	15	400	20	20	5	10	3.2
40	XAHX	5	500	20	20	3.8	10	3.2
41	YNZW	11.5	478	20	13.6	4	20	3.0
42	ZL0120	15	500	20	10	3	10	3.0
43	ZL1208	10.75	400	24	14.3	5	20	3.1
44	XAHX	15	444	20	10	3	15.3	3.2
45	YNYK	15	500	20	10	5	10	3.2
46	YNSS	5	500	20	10	5	10	3.0
47	XAHX	5	500	24	15.2	5	15.4	3.2
48	WHYC	15	500	24	10.2	3	10	3.2
49	SXAS	9	400	24	20	4.2	10	3.0
50	ZL0120	9.6	400	22.2	10	3	16.2	3.0
51	WHYC	15	400	24	10	5	10	3.0
52	YNZW	5	400	20	12.9	3	20	3.2

**Table 3:** Data blocks organization.

Blocks	Dimensions	Variables Included
**M**	11 × 9	*D_tm_*, *D_bm_*, *D_10m_*, *D_50m_*, *D_90m_*, *Span_m_*, *AOR_m_*, *HR_m_*, *SSA_m_*
**P_1_**	52 × 5	pre-mixing time (A), impeller rate (B), binder amount (C), additive rate (D), granulation time (E)
**X_2_**	52 × 9	*D_t_*, *D_b_*, *AOR*, *HR*, *MC*, *D_10_*, *D_50_*, *D_90_*, *Span*
**X_3_**	52 × 2	lubrication time (F), minimal punch tip separation distance (G)
**Y**	52 × 2	*DT*, *TS*

**Table 4:** Variance inflation factor (VIF) calculated for variables in each data block.

Data Block	Variable	VIF
**M**	*D_bm_*	8.906 × 10^3^
	*D_tm_*	6.016 × 10^3^
	*HR_m_*	353.1
	*SSA_m_*	22.09
	*AOR_m_*	5.026
	*D_10m_*	32.37
	*D_50m_*	1.338 × 10^3^
	*D_90m_*	1.554 × 10^3^
	*Span_m_*	72.03
**P_1_**	Pre-mixing time (A)	1.250
	Impeller rate (B)	1.554
	binder amount (C)	3.429
	liquid additive rate (D)	1.457
	granulation time (E)	1.632
**X_2_**	*MC*	1.611
	*AOR*	1.520
	*Db*	520.4
	*Dt*	467.0
	*HR*	118.2
	*D_10_*	13.39
	*D_50_*	394.5
	*D_90_*	501.0
	*Span*	48.36
**X_3_**	Lubrication time (F)	1.228
	Minimal punch tip separation distance (G)	7.101
**Y**	*TS*	1.006
	*DT*	1.006

**Table 5:** Diagnostics of the principal component analysis (PCA) model on data Block **M**.

PCs	Eigenvalues	*R*^2^ (%)	*R*^2^*_cum_* (%)
1	5.65	62.8	26.8
2	1.78	19.8	82.6
3	0.967	10.7	93.3

**Table 6:** Diagnostics of the PCA model on data block [**P_1_**, **X_2_**].

PCs	Eigenvalues	*R*^2^ (%)	*R*^2^*_cum_* (%)
1	4.52	32.3	32.3
2	2.40	17.1	49.4
3	1.51	10.8	60.2
4	1.16	8.3	68.5
5	1.01	7.1	75.6
6	0.94	6.7	82.3

**Table 7:** Diagnostics of the PCA model on data block [**X_3_**, **Y**].

PCs	Eigenvalues	*R*^2^ (%)	*R*^2^*_cum_* (%)
1	1.73	43.3	43.3
2	1.41	35.3	78.6
3	0.67	16.7	95.3

**Table 8:** Diagnostics of PLS, MBPLS and MBPLSPM models on the PNS IRT process.

Model	LVs	*R*^2^*_Xcum_* (%)	*R*^2^*_Ycum_* (%)	*Q*^2^*_Ycum_* (%)
PLS	3	47.0	76.1	62.5
MBPLS	3	47.4	77.7	70.8
MBPLSPM	3	64.3	79.8	63.5

## Supplementary Materials

The following are available online at https://www.mdpi.com/1999-4923/11/9/474/s1, Methods—Properties of Input Materials, Granules and Tablets; Table S1: The properties of different batches of PNS materials; Table S2: The properties of different batches of granules and tablets.

## References

[1] Yu L.X., Kopcha M. (2017). The future of pharmaceutical quality and the path to get there. Int. J. Pharm..

[2] Eberle L.G., Sugiyama H., Schmidt R. (2014). Improving lead time of pharmaceutical production processes using Monte Carlo simulation. Comput. Chem. Eng..

[3] García-Muñoz S., Dolph S., Ward H.W. (2010). Handling uncertainty in the establishment of a design space for the manufacture of a pharmaceutical product. Comput. Chem. Eng..

[4] Group I.E.W. (2008). Pharmaceutical Development Q8(R2). https://www.ich.org/products/guidelines/quality/article/quality-guidelines.html.

[5] Yu L.X., Amidon G., Khan M.A., Hoag S.W., Polli J., Raju G.K., Woodcock J. (2014). Understanding pharmaceutical quality by design. AAPS J..

[6] Norioka T., Kikuchi S., Onuki Y., Takayama K., Imai K. (2011). Optimization of the Manufacturing Process for Oral Formulations Using Multivariate Statistical Methods. J. Pharm. Innov..

[7] MacGregor J.F., Bruwer M.-J. (2008). A Framework for the Development of Design and Control Spaces. J. Pharm. Innov..

[8] Souihi N., Josefson M., Tajarobi P., Gururajan B., Trygg J. (2013). Design Space Estimation of the Roller Compaction Process. Ind. Eng. Chem. Res..

[9] Djuris J., Djuric Z. (2017). Modeling in the quality by design environment: Regulatory requirements and recommendations for design space and control strategy appointment. Int. J. Pharm..

[10] Chatzizaharia K.A., Hatziavramidis D.T. (2015). Dissolution Efficiency and Design Space for an Oral Pharmaceutical Product in Tablet Form. Ind. Eng. Chem. Res..

[11] Facco P., Dal Pastro F., Meneghetti N., Bezzo F., Barolo M. (2015). Bracketing the Design Space within the Knowledge Space in Pharmaceutical Product Development. Ind. Eng. Chem. Res..

[12] Veliz Moraga S., Villa M.P., Bertín D.E., Cotabarren I.M., Piña J., Pedernera M., Bucalá V. (2015). Fluidized-bed melt granulation: The effect of operating variables on process performance and granule properties. Powder Technol..

[13] Lepore J., Spavins J. (2008). PQLI Design Space. J. Pharm. Innov..

[14] Thirunahari S., Chow P.S., Tan R.B.H. (2011). Quality by Design (QbD)-Based Crystallization Process Development for the Polymorphic Drug Tolbutamide. Cryst. Growth Des..

[15] Portillo P.M., Ierapetritou M., Tomassone S., Mc Dade C., Clancy D., Avontuur P.P.C., Muzzio F.J. (2008). Quality by Design Methodology for Development and Scale-up of Batch Mixing Processes. J. Pharm. Innov..

[16] Prawang P., Zhang Y., Zhang Y., Wang H. (2019). Ultrasonic Assisted Extraction of Artemisinin from Artemisia Annua L. Using Poly(Ethylene Glycol): Toward a Greener Process. Ind. Eng. Chem. Res..

[17] Xie X., Schenkendorf R. (2019). Stochastic back-off-based robust process design for continuous crystallization of ibuprofen. Comput. Chem. Eng..

[18] Simonoska Crcarevska M., Geskovski N., Calis S., Dimchevska S., Kuzmanovska S., Petrusevski G., Kajdzanoska M., Ugarkovic S., Goracinova K. (2013). Definition of formulation design space, in vitro bioactivity and in vivo biodistribution for hydrophilic drug loaded PLGA/PEO-PPO-PEO nanoparticles using OFAT experiments. Eur. J. Pharm. Sci..

[19] Diab S., McQuade D.T., Gupton B.F., Gerogiorgis D.I. (2019). Process Design and Optimization for the Continuous Manufacturing of Nevirapine, an Active Pharmaceutical Ingredient for HIV Treatment. Org. Process Res. Dev..

[20] Wiest J., Saedtler M., Balk A., Merget B., Widmer T., Bruhn H., Raccuglia M., Walid E., Picard F., Stopper H. (2017). Mapping the pharmaceutical design space by amorphous ionic liquid strategies. J. Control. Release.

[21] Induru J. (2012). Excipient screening and development of formulation design space for diclofenac sodium fast dissolving tablets. Int. J. Pharm. Pharm. Sci..

[22] Jiang C., Flansburg L., Ghose S., Jorjorian P., Shukla A.A. (2010). Defining process design space for a hydrophobic interaction chromatography (HIC) purification step: Application of quality by design (QbD) principles. Biotechnol. Bioeng..

[23] Harms J., Wang X., Kim T., Yang X., Rathore A.S. (2008). Defining process design space for biotech products: Case study of Pichia pastoris fermentation. Biotechnol. Prog..

[24] Abu-Absi S.F., Yang L., Thompson P., Jiang C., Kandula S., Schilling B., Shukla A.A. (2010). Defining process design space for monoclonal antibody cell culture. Biotechnol. Bioeng..

[25] Xu B., Shi X.Y., Qiao Y.J., Wu Z.S., Lin Z.Z. (2013). Establishment of design space for production process of traditional Chinese medicine preparation. China J. Chin. Mater. Med..

[26] Gong X., Li Y., Guo Z., Qu H. (2014). Control the effects caused by noise parameter fluctuations to improve pharmaceutical process robustness: A case study of design space development for an ethanol precipitation process. Sep. Purif. Technol..

[27] Van Bockstal P.J., Mortier S., Corver J., Nopens I., Gernaey K.V., De Beer T. (2017). Quantitative risk assessment via uncertainty analysis in combination with error propagation for the determination of the dynamic Design Space of the primary drying step during freeze-drying. Eur. J. Pharm. Biopharm..

[28] Mortier S., Van Bockstal P.J., Corver J., Nopens I., Gernaey K.V., De Beer T. (2016). Uncertainty analysis as essential step in the establishment of the dynamic Design Space of primary drying during freeze-drying. Eur. J. Pharm. Biopharm..

[29] Bano G., Facco P., Ierapetritou M., Bezzo F., Barolo M. (2019). Design space maintenance by online model adaptation in pharmaceutical manufacturing. Comput. Chem. Eng..

[30] Rajalahti T., Kvalheim O.M. (2011). Multivariate data analysis in pharmaceutics: A tutorial review. Int. J. Pharm..

[31] Kayrak-Talay D., Dale S., Wassgren C., Litster J. (2013). Quality by design for wet granulation in pharmaceutical processing: Assessing models for a priori design and scaling. Powder Technol..

[32] Montes F.C.C., Gernaey K., Sin G. (2018). Dynamic Plantwide Modeling, Uncertainty, and Sensitivity Analysis of a Pharmaceutical Upstream Synthesis: Ibuprofen Case Study. Ind. Eng. Chem. Res..

[33] Tomba E., Facco P., Bezzo F., Barolo M. (2013). Latent variable modeling to assist the implementation of Quality-by-Design paradigms in pharmaceutical development and manufacturing: A review. Int. J. Pharm..

[34] Uehara N., Hayashi Y., Mochida H., Otoguro S., Onuki Y., Obata Y., Takayama K. (2016). Latent structure analysis in the pharmaceutical process of tablets prepared by wet granulation. Drug Dev. Ind. Pharm..

[35] García-Muñoz S., Polizzi M. (2012). WSPLS—A new approach towards mixture modeling and accelerated product development. Chemom. Intell. Lab. Syst..

[36] Yacoub F., Lautens J., Lucisano L., Banh W. (2011). Application of Quality by Design Principles to Legacy Drug Products. J. Pharm. Innov..

[37] Kikuchi S., Onuki Y., Yasuda A., Hayashi Y., Takayama K. (2011). Latent structure analysis in pharmaceutical formulations using Kohonen’s self-organizing map and a Bayesian network. J. Pharm. Sci..

[38] Bano G., Wang Z., Facco P., Bezzo F., Barolo M., Ierapetritou M. (2018). A novel and systematic approach to identify the design space of pharmaceutical processes. Comput. Chem. Eng..

[39] Westerhuis J.A., Smilde A.K. (2001). Deflation in multiblock PLS. J. Chemom..

[40] Troup G.M., Georgakis C. (2013). Process systems engineering tools in the pharmaceutical industry. Comput. Chem. Eng..

[41] Wang Z., Escotet-Espinoza M.S., Ierapetritou M. (2017). Process analysis and optimization of continuous pharmaceutical manufacturing using flowsheet models. Comput. Chem. Eng..

[42] Taipale-Kovalainen K., Karttunen A.P., Ketolainen J., Korhonen O. (2018). Lubricant based determination of design space for continuously manufactured high dose paracetamol tablets. Eur. J. Pharm. Sci..

[43] Pasquini C. (2018). Near infrared spectroscopy: A mature analytical technique with new perspectives—A review. Anal. Chim. Acta.

[44] Yu L.X. (2008). Pharmaceutical quality by design: Product and process development, understanding, and control. Pharm. Res..

[45] Sun F., Xu B., Zhang Y., Dai S., Yang C., Cui X., Shi X., Qiao Y. (2016). Statistical modeling methods to analyze the impacts of multiunit process variability on critical quality attributes of Chinese herbal medicine tablets. Drug Des. Dev. Ther..

[46] Chong I.-G., Jun C.-H. (2005). Performance of some variable selection methods when multicollinearity is present. Chemom. Intell. Lab. Syst..

[47] Smilde A.K., Westerhuis J.A., de Jong S. (2003). A framework for sequential multiblock component methods. J. Chemom..

[48] Bergund A., Wold S. (1999). A serial extension of MBPLS. J. Chemom..

[49] Wangen L.E., Kowalski B.R. (1988). A MBPLS algorithm investigating complex chemical systems. J. Chemom..

[50] Nomikos P., MacGregor J.F. (1995). Multivariate SPC Charts for Monitoring Batch Processes. Technometrics.

[51] Xu B., Lin Z., Wu Z., Shi X., Qiao Y., Luo G. (2013). Target-oriented overall process optimization (TOPO) for reducing variability in the quality of herbal medicine products. Chemom. Intell. Lab. Syst..

[52] Liu Z., Bruwer M.-J., MacGregor J.F., Rathore S.S.S., Reed D.E., Champagne M.J. (2011). Modeling and Optimization of a Tablet Manufacturing Line. J. Pharm. Innov..

