# Supplementary material for: A Novel Framework to Aid the Development of Design Space across Multi-Unit Operation Pharmaceutical Processes—A Case Study of Panax Notoginseng Saponins Immediate Release Tablet
Source: Pharmaceutics. 2019 Sep 13;11(9):474. doi: 10.3390/pharmaceutics11090474 (PMC6781312; doi:10.3390/pharmaceutics11090474)
Supplement: Supplementary file 1 [file pharmaceutics-11-00474-s001.pdf]

pharmaceutics

Supplementary Materials: A Novel Framework to Aid the Development of Design Space Across Multi-Unit Operation Pharmaceutical Processes—A Case Study of *Panax Notoginseng* Saponins Immediate Release Tablet

Fei Sun, Bing Xu, Shengyun Dai, Yi Zhang, Zhaozhou Lin and Yanjiang Qiao

Methods—Properties of Input Materials, Granules and Tablets

The bulk density (D_b) and the tapped density (D_t) of materials and granules were measured in a 250 mL cylinder according to the USP 39-NF34, and each batch of samples was tested in triplicate.

The Hausner ratio (*HR*) was calculated as Equation (S1).

$$HR = D_t/D_b \quad \text{(S1)}$$

Statistical parameters of the granule size distribution including D_{10}, D_{50}, and D_{90} were measured by the laser diffraction instrument (BT 2001; Dandong Bettersize Instrument Ltd., Dandong, People's Republic of China) three times. The width of the granule size distribution (ie, *span*) was calculated by Equation (S2).

$$Span = (D_{90} - D_{10})/D_{50} \quad \text{(S2)}$$

The angle of repose (*AOR*) of materials and granules was measured with the powder flow ability tester (Copley BEP2; Copley Scientific Limited, Nottingham, UK). Samples were poured through a vibrating metal funnel onto a platform until a stable and height-fixed heap was formed. The AOR was measured as the angle made by the inclined plane of the heap with the horizontal. Each batch of the sample was tested in triplicate.

The specific surface area (*SSA*) of materials was measured by applying a 3H-2000 PS1 specific surface & pore size analysis instrument (Beishide Instrument Technology Co. Ltd., Beijing, R.P China). Samples were weighed and were then poured into the sample tubes with filler rods. Before measurement, samples were degassed at 120 °C for 2.5 h to keep the surface of samples clean enough. Partial pressure points for nitrogen at 77 K were set between 0.05 and 0.3 Bar, split equally into 6 points and the equilibration time was 10 s. The SAA of materials was determined based on the Brunauer-Emmett-Teller adsorption isotherm analysis.

The moisture content (MC) of granule was tested using the Sartorius MA35 instrument (Sartorius AG, Goettingen, Germany). The sample was placed on the pan and heated to 105 °C. The percentage of moisture content was recorded until the weight was constant. Each batch of samples was tested in triplicate.

The hardness of the tablet was evaluated with the HC 97 instrument (Kraemer Elektronik GmbH, Darmstadt, Germany). The tablet diameter and thickness were measured by the digimatic indicator (ID-C112X/1012X; Mitutoyo Corporation, Kawasaki, Japan). With these obtained data, the tensile strength (*TS*) of tablets was calculated according to the equation described by Fell and Newton.

$$TS = 2F/\pi dt \quad \text{(S3)}$$

where F refers to the hardness of tablets (N), d refers to tablet diameter (mm), and t refers to tablet thickness (mm). Each batch of samples was tested 10 times.

The disintegration time was tested according to the USP 39-NF34, and each batch of samples was tested in six times.

The results of properties of input materials, granules and tablets were showed in Table S1 and Table S2.

Table S1. The properties of different batches of PNS materials.

Lots	D_{10m} (μm)	D_{50m} (μm)	D_{90m} (μm)	$Span_m$	SSA(m^2·g^{-1})	D_{bm} (g·mL^{-1})	D_{tm} (g·mL^{-1})	AOR_m (°)	HR_m
ZL1208	3.067	20.02	83.10	3.996	0.9152	0.477	0.744	47.3	1.56
ZL0120	3.829	26.51	95.51	3.457	0.6896	0.488	0.729	45.8	1.49
ZL0518	4.193	30.14	115.3	3.688	0.6891	0.507	0.755	45.2	1.49
ZL0524	3.922	28.61	102.5	3.446	0.6368	0.511	0.751	45.5	1.47
BCTG	3.792	37.75	133.6	3.438	0.6209	0.572	0.813	48.6	1.42
YNSS	3.448	30.03	112.1	3.619	0.9789	0.524	0.778	44.9	1.48
SXAS	2.107	8.518	23.72	2.536	1.512	0.387	0.587	51.0	1.52
XAHX	2.927	19.85	84.82	4.125	0.9495	0.476	0.728	50.4	1.53
WHYC	3.027	18.86	76.92	3.916	0.7675	0.318	0.506	44.7	1.59
YNZW	2.730	12.74	47.59	3.523	0.8795	0.411	0.613	44.7	1.49
YNYK	3.447	24.33	96.34	3.819	0.7176	0.488	0.752	45.7	1.54

Table S2. The properties of different batches of granules and tablets.

Runs	Granule									Tablet	
	D_b (g·mL^{-1})	D_t (g·mL^{-1})	*MC* (%)	*AOR* (°)	*HR*	D_{10} (μm)	D_{50} (μm)	D_{90} (μm)	*Span*	*TS* (Mpa)	*DT* (min)
1	1.06	32.4	0.410	0.491	1.20	38.50	120.9	249.2	1.743	3.83	2.25
2	1.70	34.0	0.389	0.474	1.19	40.82	128.7	275.8	1.827	5.08	6.01
3	1.86	34.7	0.383	0.464	1.21	16.97	66.82	164.2	2.200	6.73	5.33
4	1.93	33.0	0.479	0.547	1.14	69.18	170.5	346.5	1.627	3.67	4.87
5	1.34	33.5	0.436	0.527	1.21	61.40	141.9	287.8	1.596	3.92	6.93
6	1.50	32.0	0.410	0.475	1.16	36.85	115.6	236.8	1.729	4.88	3.17
7	1.26	32.0	0.399	0.479	1.20	36.23	109.5	219.3	1.672	4.98	3.53
8	1.22	33.0	0.450	0.541	1.20	54.60	140.5	298.4	1.735	3.87	3.34
9	1.11	32.0	0.473	0.578	1.22	66.17	145.3	294.8	1.574	1.73	6.01
10	1.55	33.0	0.486	0.583	1.20	60.01	141.4	296.9	1.675	2.45	7.41
11	1.49	33.7	0.436	0.507	1.16	56.95	228.0	552.4	2.174	3.38	4.14
12	1.21	31.6	0.467	0.569	1.22	59.50	146.8	311.2	1.715	3.14	3.51
13	1.74	33.4	0.412	0.509	1.24	29.94	98.79	210.8	1.830	4.26	2.44
14	1.75	32.9	0.456	0.540	1.19	73.58	196.1	487.4	2.110	3.96	5.41
15	1.83	33.5	0.414	0.496	1.20	44.00	171.8	460.9	2.430	5.74	5.25
16	1.92	33.2	0.491	0.561	1.14	48.91	197.4	493.6	2.253	1.94	3.81
17	1.68	33.5	0.491	0.550	1.12	61.80	222.6	538.5	2.14	2.73	7.42
18	1.87	34.3	0.409	0.467	1.14	44.19	135.1	304.4	1.927	4.98	3.94
19	1.61	32.7	0.431	0.535	1.24	72.75	236.2	549.9	2.042	3.68	4.51
20	1.07	33.3	0.453	0.557	1.23	56.69	141.7	310.6	1.792	2.85	6.54
21	1.76	33.5	0.451	0.531	1.18	57.83	141.6	299.4	1.706	2.81	3.67
22	2.04	32.6	0.472	0.540	1.14	62.24	155.3	336.9	1.77	3.45	4.80
23	1.76	32.2	0.445	0.511	1.15	46.24	134.8	292.9	1.830	4.57	3.00
24	1.64	33.4	0.445	0.507	1.14	53.03	145.6	315.7	1.804	5.04	5.39
25	1.87	34.2	0.419	0.509	1.22	22.03	81.97	210.4	2.302	4.26	3.97
26	1.55	33.3	0.447	0.549	1.23	58.40	144.3	315.6	1.782	1.98	3.39
27	1.48	34.1	0.455	0.540	1.19	67.14	155.0	322.7	1.649	3.99	5.68
28	1.97	33.1	0.443	0.523	1.18	52.41	142.4	309.6	1.806	4.07	2.90
29	1.80	33.9	0.464	0.526	1.13	53.16	143.0	310.9	1.802	4.78	4.13
30	1.85	33.1	0.452	0.544	1.20	57.48	142.7	301.7	1.712	3.64	4.82
31	1.77	33.7	0.461	0.570	1.24	68.6	155.9	322.8	1.631	2.11	4.26
32	1.88	33.8	0.436	0.543	1.25	59.78	147.3	311.9	1.712	4.22	5.87
33	1.75	33.2	0.442	0.515	1.16	31.99	108.1	238.1	1.906	4.14	3.47
34	1.71	32.2	0.487	0.558	1.15	73.59	167.0	339.4	1.592	2.94	5.81
35	1.29	34.3	0.436	0.522	1.20	55.02	138.1	294.6	1.735	3.56	3.40
36	1.47	33.6	0.427	0.535	1.25	58.44	145.3	307.8	1.716	3.09	3.20
37	1.87	34.3	0.440	0.501	1.14	65.49	171.5	400.7	1.953	5.50	6.20
38	1.63	33.8	0.392	0.487	1.24	47.48	133.0	291.2	1.833	4.73	3.03
39	1.63	32.1	0.428	0.502	1.17	48.39	138.0	306.8	1.872	4.37	3.11
40	1.86	33.1	0.425	0.496	1.17	42.33	129.2	289.2	1.910	3.93	2.11
41	1.67	34.1	0.408	0.484	1.19	35.93	113.6	254.4	1.922	5.48	3.78
42	1.64	33.1	0.406	0.506	1.25	46.91	125.9	274.9	1.811	5.18	3.71
43	1.52	34.4	0.447	0.531	1.19	65.02	160.1	333.1	1.674	3.60	5.37
44	2.10	34.0	0.416	0.480	1.15	38.43	117.4	264.3	1.924	5.22	2.40
45	1.49	33.1	0.441	0.529	1.20	54.73	147.3	319.2	1.796	3.78	2.78
46	1.27	33.0	0.442	0.539	1.22	54.84	148.4	319.8	1.786	4.67	3.52
47	1.70	33.2	0.477	0.554	1.16	64.85	163.7	335.8	1.655	2.96	4.62
48	1.55	34.1	0.442	0.533	1.21	69.01	153.3	314.4	1.601	3.12	4.65
49	1.54	33.1	0.430	0.504	1.17	41.10	134.0	310.0	2.077	6.07	7.06
50	1.22	33.5	0.456	0.538	1.18	61.51	150.1	316.0	1.695	3.28	7.66
51	1.65	32.4	0.469	0.535	1.14	74.40	211.4	516.4	2.094	4.09	7.77
52	1.64	34.1	0.394	0.466	1.18	35.90	122.1	291.3	2.092	3.90	2.76
